# Establishing an early warning alert and response network following the Solomon Islands tsunami in 2013

**DOI:** 10.2471/BLT.13.133512

**Published:** 2014-08-15

**Authors:** Augustine Bilve, Francisco Nogareda, Cynthia Joshua, Lester Ross, Christopher Betcha, Kara Durski, Juliet Fleischl, Eric Nilles

**Affiliations:** a Ministry of Health and Medical Services, Lata, Temotu Province, Solomon Islands.; b Emerging Disease Surveillance and Response, World Health Organization, Providence Plaza (Level 4), Downtown Boulevard, Suva, Fiji.; c Ministry of Health and Medical Services, Honiara, Solomon Islands.; d World Health Organization, Honiara, Solomon Islands.

## Abstract

**Problem:**

On 6 February 2013, an 8.0 magnitude earthquake generated a tsunami that struck the Santa Cruz Islands, Solomon Islands, killing 10 people and displacing over 4700.

**Approach:**

A post-disaster assessment of the risk of epidemic disease transmission recommended the implementation of an early warning alert and response network (EWARN) to rapidly detect, assess and respond to potential outbreaks in the aftermath of the tsunami.

**Local setting:**

Almost 40% of the Santa Cruz Islands’ population were displaced by the disaster, and living in cramped temporary camps with poor or absent sanitation facilities and insufficient access to clean water. There was no early warning disease surveillance system.

**Relevant changes:**

By 25 February, an EWARN was operational in five health facilities that served 90% of the displaced population. Eight priority diseases or syndromes were reported weekly; unexpected health events were reported immediately. Between 25 February and 19 May, 1177 target diseases or syndrome cases were reported. Seven alerts were investigated. No sustained transmission or epidemics were identified. Reporting compliance was 85%. The EWARN was then transitioned to the routine four-syndrome early warning disease surveillance system.

**Lesson learnt:**

It was necessary to conduct a detailed assessment to evaluate the risk and potential impact of serious infectious disease outbreaks, to assess whether and how enhanced early warning disease surveillance should be implemented. Local capacities and available resources should be considered in planning EWARN implementation. An EWARN can be an opportunity to establish or strengthen early warning disease surveillance capabilities.

## Résumé

**Problème:**

Le 6 février 2013, un séisme de magnitude 8,0 a provoqué un tsunami qui a frappé les Îles Santa Cruz, aux Îles Salomon, tuant 10 personnes et déplaçant plus de 4700 autres.

**Approche:**

Une évaluation après la catastrophe du risque de transmission de maladies épidémiques a recommandé la mise en œuvre d'un réseau d'alerte et d'intervention rapides (EWARN) pour détecter, évaluer et répondre rapidement aux épidémies potentielles pouvant survenir après le tsunami.

**Environnement local:**

Près de 40% de la population des Îles Santa Cruz a été déplacée par la catastrophe et a vécu dans des camps temporaires exigus avec de mauvaises installations sanitaires, voire sans installation sanitaire, et un accès insuffisant à l'eau potable. Aucun système de surveillance des maladies et d'alerte rapide n'existait au préalable.

**Changements significatifs:**

Le 25 février, un EWARN était opérationnel dans les 5 établissements de santé qui desservaient 90% de la population déplacée. Huit maladies ou syndromes prioritaires étaient signalés chaque semaine et les cas de décès imprévus étaient signalés immédiatement. Entre le 25 février et le 19 mai, 1777 cas de maladies ou syndromes cibles ont été signalés. Sept alertes ont fait l'objet d'une enquête. Aucune transmission ou épidémie durable n'a été identifiée. La conformité des signalements était de 85%. L'EWARN est ensuite passé ensuite au système de routine de surveillance des maladies et d'alerte rapide pour quatre syndromes.

**Leçons tirées:**

Il était nécessaire de mener une analyse détaillée pour évaluer le risque et l'impact potentiel des épidémies de maladies infectieuses graves, pour évaluer si et de quelle façon un système de surveillance des maladies et d'alerte rapide devait être mis en œuvre. Les capacités locales et les ressources disponibles doivent être prises en compte dans la planification de la mise en place de l'EWARN. Un EWARN peut être l’occasion d’établir ou de renforcer les capacités de surveillance des maladies et d'alerte rapide.

## Resumen

**Situacíon:**

El 6 de febrero de 2013, un terremoto de magnitud de 8,0 dio lugar a un tsunami que azotó las islas Santa Cruz, en las Islas Salomón, que causó la muerte de 10 personas y desplazó a más de 4700.

**Enfoque:**

Una evaluación posterior a la catástrofe sobre el riesgo de transmisión de enfermedades epidémicas recomienda la aplicación de una red de alerta y respuesta temprana (EWARN) a fin de detectar, evaluar y responder rápidamente a los posibles brotes de enfermedades a raíz del tsunami.

**Marco regional:**

La catástrofe desplazó a casi el 40% de la población de las islas de Santa Cruz, la cual vive en campamentos temporales abarrotados con instalaciones sanitarias deficientes o inexistentes e insuficiente acceso al agua potable. No se disponía de un sistema de vigilancia de enfermedades para la alerta temprana.

**Cambios importantes:**

El 25 de febrero, un EWARN entró en funcionamiento en cinco centros de salud que atendían al 90% de la población desplazada. Cada semana se notificaron ocho enfermedades o síndromes prioritarios, por otro lado, los eventos de salud inesperados se comunicaron de inmediato. Entre el 25 de febrero y el 19 de mayo, se notificaron 1177 casos de enfermedades o síndromes diana. Se examinaron siete alertas. No se identificaron transmisiones o epidemias prolongadas. El cumplimiento de las notificaciones fue del 85%. Posteriormente, el EWARN se transfirió al sistema de vigilancia sistemática de enfermedades para la alerta temprana de cuatro síndromes.

**Lecciones aprendidas:**

Fue necesario realizar una evaluación completa para evaluar el riesgo y el impacto posible de brotes de enfermedades infecciosas graves, así como evaluar si y cómo debe aplicarse una mejor vigilancia de enfermedades para la alerta temprana. Debe considerarse la capacidad local y los recursos disponibles en la planificación de la ejecución del EWARN. Un EWARN puede representar una oportunidad para establecer o reforzar la capacidad de vigilancia de enfermedades para la alerta temprana.

## ملخص

**المشكلة:**

في 6 شباط/ فبراير 2013، تسبب زلزال قوته8.0 درجات في أمواج تسونامي ضربت جزر سانتا كروز وجزر سولومون وأسفرت عن مقتل 10 أشخاص وتشريد ما يزيد عن 4700 شخص.

**الأسلوب:**

أوصى تقييم ما بعد الكارثة بشأن خطورة سريان الأمراض الوبائية بتنفيذ شبكة الإنذار المبكر والمواجهة (EWARN) من أجل كشف الفاشيات المحتملة في أعقاب أمواج تسونامي وتقييمها ومواجهتها بشكل سريع.

**المواقع المحلية:**

أدت الكارثة إلى تشريد 40 % تقريباً من سكان جزر سانتا كروز وإقامتهم في مخيمات مؤقتة ضيقة مع الافتقار إلى مرافق الإصحاح أو ضعفها وعدم كفاية الوصول إلى المياه النقية. ولم يكن هناك نظام لترصد الأمراض والإنذار المبكر.

**التغيّرات ذات الصلة:**

بحلول 25 شباط/ فبراير، تم تشغيل شبكة الإنذار المبكر والمواجهة في خمسة مرافق صحية خدمت 90 % من السكان المشردين. وتم الإبلاغ عن ثمانية أمراض أو متلازمات ذات أولوية؛ وتم الإبلاغ عن الأحداث الصحية غير المتوقعة على الفور. وفي الفترة من 25 شباط/ فبراير إلى 19 أيار/ مايو، تم الإبلاغ عن 1177 حالة أمراض أو متلازمة مستهدفة. وتم تحري سبعة إنذارات. ولم يتم تحديد سريان أو أوبئة على نحو مستدام. وكانت نسبة الإبلاغ عن الامتثال 85 %. وتم تحويل شبكة الإنذار المبكر والمواجهة عندئذ إلى نظام لترصد الأمراض والإنذار المبكر الروتيني لأربع متلازمات.

**الدروس المستفادة:**

كان من الضروري إجراء تقييم مفصل لتقدير خطورة فاشيات الأمراض المعدية الخطيرة وأثرها المحتمل، وتقييم ما إذا كان من اللازم تنفيذ الترصد المحسن للأمراض في مرحلة الإنذار المبكر وكيفية ذلك. ينبغي التفكير في القدرات المحلية والموارد المتاحة لدى تخطيط تنفيذ شبكة الإنذار المبكر والمواجهة. ومن الممكن أن تمثل شبكة الإنذار المبكر والمواجهة فرصة لإنشاء قدرات ترصد الأمراض والإنذار المبكر أو دعمها.

## 摘要

**问题:**

2013年2月6日8.0级地震产生的海啸袭击了所罗门群岛的圣克鲁斯群岛，造成10人死亡，超过4700人流离失所。

**方法:**

灾后疫病传播的风险评估建议实施预警警报和响应网络（EWARN）以快速检测、评估和应对可能爆发海啸的后果。

**当地状况:**

几乎40%的圣克鲁斯群岛人口因为灾难流离失所，生活在狭小的临时营地里，这些营地缺少卫生设施或其设施条件很差，无法提供足够的干净水源。当时也没有疾病预警监测系统。

**相关变化:**

截至2月25日，已在五个卫生设施运行了一个EWARN，这些设施为90%流离失所的人口服务。每周报告八种重点疾病或并发症；立即报告突发卫生事件。在2月25日至5月19日之间，报告了1177例目标疾病或并发症病例。对七次警报进行了调查。未发现持续传播和流行传染病。报告依从性为85%。而后EWARN过渡到常规四种并发症预警疾病监测系统。

**经验教训:**

有必要进行详细的评估来评价严重的传染性疾病暴发的风险和潜在影响，并评估是否应该以及如何实施强化的预警疾病监测。在规划EWARN实施时应考虑当地的能力和可用的资源。EWARN可能是建立或强化疾病预警监测能力的机会。

## Резюме

**Проблема:**

Землетрясение магнитудой 8,0 балла, произошедшее 6 февраля 2013 года, вызвало цунами, которое обрушилось на острова Санта-Крус и Соломоновы Острова, в результате чего погибло 10 человек и осталось без крова более 4700 человек.

**Подход:**

По результатам оценки риска распространения эпидемии заболеваний было рекомендовано реализовать систему раннего предупреждения и ответных мер (EWARN) с целью быстрого выявления, оценки и реагирования на потенциальные вспышки заболеваний в зоне ликвидации последствий цунами.

**Местные условия:**

Почти 40% населения островов Санта-Крус осталось без крова в результате стихийного бедствия и живет в стесненных временных лагерях с плохими или отсутствующими санитарно-техническими сооружениями и недостаточным наличием чистой воды. При этом отсутствовала система раннего предупреждения эпиднадзора.

**Осуществленные перемены:**

По состоянию на 25 февраля система EWARN действовала в пяти медицинских учреждениях, которые обслуживали 90% живущего во временных лагерях населения. В рамках этой системы еженедельно сообщалось о восьми приоритетных заболеваниях или синдромах; о неожиданных событиях, связанных со здоровьем, сообщалось немедленно. В период с 25 февраля по 19 мая было сообщено о 1177 заболеваниях или синдромах, за которыми велось наблюдение. Семь из этих предупреждений было исследовано. При этом не было выявлено ни одного случая устойчивой передачи заболевания или эпидемии. Выполнение требований по отправке предупреждений составило 85%. Затем система EWARN была преобразована в систему раннего предупреждения текущего эпиднадзора на основе четырех симптомов.

**Выводы:**

Потребовалась детальная оценка степени риска и потенциального воздействия серьезных вспышек инфекционных заболеваний, а также того, насколько необходима усиленная система раннего предупреждения и каким образом она должна быть реализована. Планируя создание системы EWARN, следует принимать во внимание местные возможности и имеющиеся ресурсы. На основе системы EWARN может быть реализована или расширена система эпиднадзора по раннему предупреждению о заболеваниях.

## Introduction

On 6 February 2013, an 8.0 magnitude earthquake generated a three-metre tsunami that struck the remote Santa Cruz Islands (population 11 578, 2009 census)^1^ in the south-eastern Solomon Islands. The tsunami destroyed or damaged 1168 homes, severely damaged or destroyed water sources, and disrupted sanitation facilities. Ten people were killed and over 4700 were displaced.^2^

Despite debate over the magnitude of epidemic risk in the post-disaster setting,^3^^–^^5^ increased communicable disease transmission has been well documented. Thus, it is recommended that a detailed assessment to quantify epidemic risk be quickly conducted, after acute life-saving and initial rapid-assessment activities. If indicated, based on the findings of the risk assessment, a post-disaster early warning alert and response network (EWARN) should be implemented to rapidly identify and control disease epidemics.

Given the potential for enhanced disease transmission, the Solomon Islands Ministry of Health and Medical Services requested assistance from the World Health Organization (WHO) to assess and, as necessary, mitigate post-disaster epidemic risk. On 19 and 20 February 2013, a joint, on-site Ministry of Health and Medical Services and WHO assessment identified multiple post-disaster conditions that increased the risk of an epidemic, and recommended implementation of a post-disaster EWARN. We describe here the implementation, monitoring and results of the Santa Cruz Islands post-disaster EWARN, and key lessons learnt.

## Approach

### Epidemic risk assessment

The post-disaster risk assessment identified several factors that increased the risk of epidemic disease transmission. These included limited access to clean water, poor sanitation, high population density in displaced camps, living in bush, scrub and muddy environments, and increased exposure to disease vectors.^6^ The increased risk of an epidemic and the absence of early warning disease surveillance were the key findings when considering EWARN implementation.

### Development of strategy

Senior provincial medical and public health staff worked closely with a WHO epidemiologist to develop an EWARN strategy and implementation plan. The objective was to establish a simple and streamlined surveillance network to assure timely identification of and response to potentially serious disease epidemics. A chaotic post-disaster setting and substantially reduced resources precluded comprehensive disease monitoring. Given the local context and limitations, simplicity was considered essential for successful implementation and operation of the EWARN.

### Reporting sites

Criteria for EWARN site selection included proximity to displaced people and catchment population, and communication capabilities, including mobile phone network, long-wave radio or road connection to the EWARN coordination centre based at the provincial hospital. Five health facilities were selected: the provincial hospital, three health centres and one nursing station, all of which had reliable communication capabilities. The estimated EWARN catchment population was 8000 people, and it included 90% of the 4700 who had been displaced.

### Selection of diseases

Eight target diseases and syndromes were selected based on severity (life-threatening or potentially life-threatening), transmission potential (epidemic or possible epidemic capacity) and relevance to the post-disaster setting (present or likely present), as described in the post-disaster risk assessment.^6^ Target diseases included malaria, suspected dengue and suspected scrub typhus; target syndromes included influenza-like illness, watery diarrhoea, bloody diarrhoea, acute fever and rash, and prolonged fever. A case definition was established for all target diseases and syndromes (for more information contact author). In addition, event-based surveillance collected ad hoc information on health events or potential risks to health, including unexpected deaths, clusters of unusual or severe disease, chemical spills, and bird, fish or animal die-offs.

### Defining alerts

An alert was defined as “an increase in number of cases of a specific disease or syndrome beyond what was expected for that reporting site, based on trends in weekly target diseases and standardized thresholds.”^7^ Site-specific thresholds based on historical data could not be calculated because of the absence of pre-disaster surveillance data. Any unexpected event reported through the event-based surveillance was treated as an alert.

## Implementation

Between 21 and 24 February 2013, a joint Ministry of Health and Medical Services and WHO team implemented the EWARN. A senior public health nurse was identified as the EWARN coordinator, responsible for data collection, alert verification, monitoring and response coordination. Two-hour trainings for all Ministry of Health and Medical Services staff involved in the EWARN were conducted at each of the five EWARN sites to review the target diseases and syndromes, case definitions, criteria for unexpected events, data collection forms, reporting procedures and timelines. A local outbreak response team – comprising nursing, health promotion, logistics, and environmental health staff – was established. Also, a half-day training was conducted on EWARN alert verification, and on how to perform investigations and implement rapid control measures. Challenges to EWARN implementation were largely transportation-related; that is, they were related to difficulties in accessing the rural health facilities due to a shortage of available vehicles, competing priorities in the post-disaster phase or a lack of the fuel for the outboard motors needed to visit the northern and eastern villages and health facilities that are remote and accessible only by sea. These challenges were resolved by the provincial nursing director, the most senior Ministry of Health and Medical Services staff on the Santa Cruz Islands, who had the authority to identify and release the necessary resources.

### Data procedure

Cases that met the case definition for a target disease or syndrome were stratified by age (less than 5 years, 5–14 years and more than 14 years of age), and recorded on a tally sheet and line list. Surveillance data were communicated weekly via the EWARN coordinator to the Ministry of Health and Medical Services National Surveillance Unit in the capital Honiara, which analysed data and monitored for (and assured verification of) any alert. Any unusual or unexpected event was immediately reported to the EWARN coordinator.

### Surveillance outputs

Between 25 February and 19 May 2013 (after 12 weeks of EWARN operations), a total of 1177 target diseases or syndrome cases were registered from a total of 5323 estimated consultations. Influenza-like illness was most commonly reported (626 cases, 11.8% of consultations) followed by watery diarrhoea (171 cases, 3.2%), malaria (94 cases, 1.8%), acute fever and rash (90 cases, 1.7%), prolonged fever (90 cases, 1.7%), suspected dengue (57 cases, 1.1%), suspected scrub typhus (32 cases, 0.6%) and bloody diarrhoea (17 cases, 0.3%); the remainder of the consultations (4146, 77.9%) were for other causes.

Seven alerts were generated, verified and, as necessary, investigated: watery diarrhoea (2 alerts), bloody diarrhoea (1 alert), acute fever and rash (1 alert), prolonged fever (1 alert), suspected dengue (1 alert) and influenza-like illness (1 alert). No unusual or expected events were reported through the event-based surveillance. Alerts were verified by asking the relevant health-care providers to confirm the data and provide additional information on clinical features, disease severity, and whether the number of new cases was increasing or decreasing. Verified alerts with evidence of ongoing transmission were investigated by the EWARN outbreak response team. The dengue-like illness alert was a reporting error. Of the six verified alerts, four events were isolated and the rate of new cases returned to baseline without intervention. The two remaining alerts required the investigation team to be dispatched and control measures implemented. None of the alerts progressed to sustained transmission; therefore, no laboratory samples were collected by the investigation teams. Fifty-one weekly reports were received (51/60, 85% reporting compliance) within 24 hours of the weekly reporting deadline. The remaining nine reports (15%) were not received.

## Discussion

We implemented an EWARN that included indicator and event-based surveillance components two weeks after an earthquake and tsunami, despite limited human, medical, laboratory and logistical capacities; a remote setting; and a challenging post-disaster context. Several alerts were reported and investigated, but none led to sustained transmission. In May 2013, a repeated risk assessment found a decreased risk of epidemic transmission. On 20 May, the EWARN was transitioned to a four-syndrome early warning disease surveillance system that aligns with the Solomon Islands and Pacific Syndromic Surveillance Systems.^8^ These systems monitor influenza-like illness, diarrhoea, acute fever and rash, and prolonged fever plus any unexpected or unusual event with potential health implications.

One limitation of the EWARN was an absence of baseline surveillance data to establish alert thresholds (weekly trends and existing standard reference materials were used instead). Another limitation was that site-selection criteria may have introduced an unintended bias that systematically recruited sites at higher or lower risk of outbreaks. For example, sites and communities with reliable communication may have better water and sanitation infrastructure that would in turn systematically decrease the risk of enteric disease outbreak in those communities. Also, the EWARN did not capture all affected populations, and localized outbreaks may have been missed.

Certain post-disaster features (e.g. population displacement, crowded living conditions, limited clean water and poor sanitation) increase the risk of an epidemic.^9^^–^^14^ Hence, an assessment is necessary to quantify the risk and, as appropriate, guide mitigation interventions. Important factors when considering EWARN implementation include magnitude of epidemic risk, existing capacity to identify and respond, and available resources. Post-disaster environments are usually resource-scarce; thus, any activity should consider resource implications. Immediate post-disaster environments are typically dynamic, with ongoing population movement and changes in epidemic risk characteristics (including water quality and quantity, hygiene, sanitation and access to health care). Therefore, the timing of a risk assessment has implications for estimation of the risk of an epidemic and consideration of risk mitigation.

Given the substantial human, material and financial resources mobilized in a disaster response, it is important to link post-disaster activities to long-term development goals. EWARN systems, as one element of a disaster response, are well suited to building or strengthening sustainable early warning disease surveillance and response, and thereby help countries meet their International Health Regulation obligations to achieve and maintain essential surveillance and response capacity.^15^ As with all surveillance systems, ongoing monitoring, assessment, feedback and training is necessary to assure sustainability.

We learnt several lessons that will be useful when considering post-disaster early warning disease surveillance (Box 1). First, promptly conducting a detailed assessment to evaluate the risk and potential impact of serious infectious disease outbreaks is necessary to assess whether and how enhanced early warning disease surveillance should be implemented. Second, local capacities and available resources should be considered in planning EWARN implementation. Finally, an EWARN can be an opportunity to establish or strengthen sustainable early warning disease surveillance capabilities. No pre-earthquake disease surveillance was operational on the Santa Cruz Islands. However, the EWARN established a surveillance infrastructure and, after three months of operation, it was smoothly transitioned, with minimal additional financial or material resources, into the routine Solomon Islands Syndromic Surveillance System.

Box 1 **Summary of main lessons learnt**

- A detailed assessment to evaluate the risk and potential impact of serious infectious disease outbreaks is necessary to assess whether and how enhanced early warning disease surveillance should be implemented.
- Local capacities and available resources should be considered in planning EWARN implementation.
- An EWARN can be an opportunity to establish or strengthen early warning disease surveillance capabilities.

EWARN, early warning alert and response network.
